# Supplementary material for: Prostaglandin E2 dependent migration of human brain endothelial cells is mediated through Rho-Kinase-II
Source: PLoS One. 2025 Jun 23;20(6):e0326312. doi: 10.1371/journal.pone.0326312 (PMC12185010; doi:10.1371/journal.pone.0326312)
Supplement: Attachment — Submitted filename: Combined point to point responses.docx [file pone.0326312.s002.docx]

**Journal requirements:**

When submitting your revision, we need you to address these additional requirements.

Q1. Please ensure that your manuscript meets PLOS ONE's style requirements, including those for file naming. The PLOS ONE style templates can be found at

<https://journals.plos.org/plosone/s/file?id=wjVg/PLOSOne_formatting_sample_main_body.pdf> and <https://journals.plos.org/plosone/s/file?id=ba62/PLOSOne_formatting_sample_title_authors_affiliations.pdf>

**Response:**

Thank you for your valuable suggestions. We have revised and reorganized the entire manuscript in accordance with the PLOS ONE formatting guidelines provided in the aforementioned links.

Q2. We noticed you have some minor occurrence of overlapping text with the following previous publication(s), which needs to be addressed:

https://academic.oup.com/jleukbio/article-abstract/105/4/705/6935763?redirectedFrom=fulltext&login=false

https://d.docksci.com/post-polymerization-crosstalk-between-the-actin-cytoskeleton-and-microtubule-net_5a0eb427d64ab2eca1fa284b.html

https://www.thno.org/v08p6053.htm

In your revision ensure you cite all your sources (including your own works), and quote or rephrase any duplicated text outside the methods section. Further consideration is dependent on these concerns being addressed.

**Response:**

Thanks for the comments! We recognize that some phrasing—primarily in the background and discussion sections—may have inadvertently mirrored wording from these sources, including our own previously published material with proper references. We affirm that there was no intent to misappropriate text and that the overlap was limited to commonly used technical expressions in molecular biology. We are committed to upholding the ethical standards of scientific publishing and will resubmit the revised manuscript for transparency. Thank you for your guidance and continued consideration.

Q3. To comply with PLOS ONE submissions requirements, in your Methods section, please provide additional information regarding the experiments involving animals and ensure you have included details on (1) methods of sacrifice, and (2) efforts to alleviate suffering.

**Response:**

Thank you for your valuable suggestions. We have revised the section on animal experiments in the Materials and Methods under the heading “Animals and Ethical Considerations” [page 4, Lines 120–127]. The updated text provides a clearer and more detailed description of the experimental protocol, including ethical approval, housing conditions, and animal handling procedures, in accordance with internationally accepted guidelines. We believe these changes significantly strengthen the methodological rigor and ethical transparency of our study.

Q4. Please include a complete copy of PLOS’ questionnaire on inclusivity in global research in your revised manuscript. Our policy for research in this area aims to improve transparency in the reporting of research performed outside of researchers’ own country or community. The policy applies to researchers who have travelled to a different country to conduct research, research with Indigenous populations or their lands, and research on cultural artefacts. The questionnaire can also be requested at the journal’s discretion for any other submissions, even if these conditions are not met. Please find more information on the policy and a link to download a blank copy of the questionnaire here: https://journals.plos.org/plosone/s/best-practices-in-research-reporting. Please upload a completed version of your questionnaire as Supporting Information when you resubmit your manuscript.

**Response:**

Thank you for your valuable suggestions. However, this point is not relevant to our current study.

Q5. Thank you for stating the following in the Acknowledgments Section of your manuscript:

“This work was supported by grants DSR KFU”

We note that you have provided funding information that is not currently declared in your Funding Statement. However, funding information should not appear in the Acknowledgments section or other areas of your manuscript. We will only publish funding information present in the Funding Statement section of the online submission form.

Please remove any funding-related text from the manuscript and let us know how you would like to update your Funding Statement. Currently, your Funding Statement reads as follows:

“The author(s) received no specific funding for this work.”

Please include your amended statements within your cover letter; we will change the online submission form on your behalf.

**Response:**

Thank you for your valuable suggestions. We have changed the manuscript as per your above suggestion. We will include the funding number later if any.

Q6. We note that your Data Availability Statement is currently as follows: [All relevant data are within the manuscript and its supporting information files.]

Please confirm at this time whether or not your submission contains all raw data required to replicate the results of your study. Authors must share the “minimal data set” for their submission. PLOS defines the minimal data set to consist of the data required to replicate all study findings reported in the article, as well as related metadata and methods (https://journals.plos.org/plosone/s/data-availability#loc-minimal-data-set-definition).

For example, authors should submit the following data:

- The values behind the means, standard deviations and other measures reported;

- The values used to build graphs;

- The points extracted from images for analysis.

Authors do not need to submit their entire data set if only a portion of the data was used in the reported study.

If your submission does not contain these data, please either upload them as Supporting Information files or deposit them to a stable, public repository and provide us with the relevant URLs, DOIs, or accession numbers. For a list of recommended repositories, please see https://journals.plos.org/plosone/s/recommended-repositories.

If there are ethical or legal restrictions on sharing a de-identified data set, please explain them in detail (e.g., data contain potentially sensitive information, data are owned by a third-party organization, etc.) and who has imposed them (e.g., an ethics committee). Please also provide contact information for a data access committee, ethics committee, or other institutional body to which data requests may be sent. If data are owned by a third party, please indicate how others may request data access.

**Response:**

Thank you for your valuable suggestion. During resubmission we will share the raw data of ELISA in excel file as needed

7. PLOS ONE now requires that authors provide the original uncropped and unadjusted images underlying all blot or gel results reported in a submission’s figures or Supporting Information files. This policy and the journal’s other requirements for blot/gel reporting and figure preparation are described in detail at https://journals.plos.org/plosone/s/figures#loc-blot-and-gel-reporting-requirements and https://journals.plos.org/plosone/s/figures#loc-preparing-figures-from-image-files. When you submit your revised manuscript, please ensure that your figures adhere fully to these guidelines and provide the original underlying images for all blot or gel data reported in your submission. See the following link for instructions on providing the original image data: https://journals.plos.org/plosone/s/figures#loc-original-images-for-blots-and-gels.

**Response:**

Thank you for your suggestion. We will do the needful.

In your cover letter, please note whether your blot/gel image data are in Supporting Information or posted at a public data repository, provide the repository URL if relevant, and provide specific details as to which raw blot/gel images, if any, are not available. Email us at plosone@plos.org if you have any questions.

**Reviewer’s comments**

**Reviewer #1:**

***Comment:***

It is well structured and nicely written research work. Similar work has been done before but you have added animal study to it. The work is generally acceptable but you need to make it concise and to the point with results and findings more magnified.

***Response:***

Thank you for your valuable comments and constructive suggestions on our manuscript. We appreciate your positive feedback. In response, we have revised the manuscript to improve its clarity, conciseness, and overall focus. The edited sections have been clearly highlighted for your convenience. We have also refined the presentation of our results and findings to ensure better clarity and scientific rigor. Furthermore, we have enhanced the quality of several figures to improve readability and facilitate a clearer understanding of the data presented.

**Reviewer #2:**

In the current manuscript, the authors report that Prostaglandin E_2_ (PGE_2_) plays a crucial role in the migration of Human Brain Endothelial Cells through the activation of Rho Kinase II (ROCK-II). The paper is well written and organized. However, following are the concerns:

***Comment 1:***

In Figure 5a, the pMLC band after the addition of PGE_2_ appears thinner and weaker than the bands observed under the same conditions in Figures 5b and 5c (+PGE_2_ lanes). It is unclear why such a discrepancy occurs. Additionally, the author’s state that the membrane used to detect pMLC was reprobed to detect total MLC. However, the shapes and widths of the respective bands appear to differ (for example, in Figure 5c). If the same membrane was used for both detections, the band shapes for pMLC and MLC should be identical. If different membranes were used instead, the authors should clearly indicate this and ideally provide results obtained from the same membrane.2.

**Response 1:**

We thank the reviewer comments/concern. We have modified Figure 5a accordingly. Separate experiments were conducted for pMLC and total PMC blotting, and each was reprobed with β-Actin antibody independently as a loading control. This clarification has been incorporated into the *Materials and Methods* section under the subheading Immunoblotting (page 8, lines 236–242), as well as in the figure legend page (page 22, lines 726–731) in the revised manuscript.

***Comments 2:***

In Figure 6, the image showing actin polymerization is not very clear. In particular, in Figure 6b, the authors state that “NS-siRNA has no effect,” but the development of F-actin is not readily discernible. If a higher-quality image is available, I would suggest replacing the current one.

***Response 2:***

We thank the reviewer for their valuable observation regarding Figure 6a & 6b. To improve the clarity and visibility of F-actin polymerization, we have updated the figures with higher-magnification images in BOX. Specifically, images were originally acquired at both 20X and 40X magnification. In the revised version, we selected a representative region from the 20X image and magnified it using 40X to enhance resolution and provide a clearer visualization of F-actin structures. This adjustment should allow for better assessment of the statement that "NS-siRNA has no effect." We hope the revised images address the concern effectively. (page 22, line 750-751 and page 23 line 756-757)

***Comments 3:***

The authors performed a mouse aortic ring sprouting assay and showed that pre-treatment with Y27632 repressed angiogenic sprouting/aorta outgrowth in Figure 7.However, I was unable to understand how they reached this conclusion. Would it be possible to quantitatively analyse the results as done in the following paper? <https://doi.org/10.1016/j.mbplus.2020.100025>

***Response 3:***

We thank you the reviewer for the comments. The mouse aortic ring assay is a well-established ex vivo model that recapitulates key processes of angiogenesis, including endothelial cell proliferation, migration, and microvessel sprouting. In our study, we utilized this assay to assess angiogenic activity, employing quantification methods aligned with validated protocols from prior literature. For instance, Nunes *et al*. (2020) [DOI: 10.1016/j.mbplus.2020.100025] demonstrated that microvessel outgrowth from aortic rings reliably reflects angiogenic potential, allowing for robust quantitative analysis in response to pro- and anti-angiogenic stimuli. Similarly, the PLOS ONE study <https://journals.plos.org/plosone/article/figure?id=10.1371/journal.pone.0069552.g002>] showed that metrics such as sprout number and cumulative sprout length are sensitive and reliable indicators of angiogenic response. Consistent with these studies, we quantified microvessel sprouting using analogous parameters, including the number and total length of sprouts. This methodological consistency reinforces the validity of our findings and supports the conclusion that the observed sprouting reflects angiogenic induction. Accordingly, we have add and discussed (page 9, line 274-284.Page 14, line 411-419) and Figure 7b legend (page 23, line 767-770) & Two references were added (page 18, line 566-569 & page 20 line 633-636)

**Minor comments:**

1. The order of the samples in the PCR results is confusing (in Figure 1). Could this be corrected?

**Response 1:**

Thank you for your valuable comments. We have carefully addressed your feedback and have updated Figure 1 to reflect the correct markings and sequence. Additionally, we have included a box around the relevant area to enhance clarity and ensure the sequence is easily identifiable.

We appreciate your attention to detail and believe these revisions improve the overall presentation of the data.

2. I would like to see the units standardized. For example, “0.1 nM” in the figure and “100 pM” in the text (page 10, line 296 and 300), etc.

**Response 2:**

Thank you for taking the time to review our work and for providing detailed and insightful comments. We have addressed the unit discrepancy between the Results section and the figure by standardizing all relevant units to "nM." These revisions have been implemented in the updated manuscript, specifically (page 11, lines 317 and 321).

3. There is no Figure 3c, but the text refers to Fig 3c in page 11 (is it a mistake for Fig 3b?).

**Response 3:**

Thank you for your careful review of our manuscript and for pointing out the error in Figure 3. We appreciate your attention to detail. You are correct that it was incorrectly labelled as 3c instead of 3b. This mistake has been rectified in the revised manuscript, as reflected (page 12, line 343-344).
