# Supplementary material for: Prostaglandin E2 dependent migration of human brain endothelial cells is mediated through Rho-Kinase-II
Source: PLoS One. 2025 Jun 23;20(6):e0326312. doi: 10.1371/journal.pone.0326312 (PMC12185010; doi:10.1371/journal.pone.0326312)
Supplement: Attachment — Submitted filename: Point to point responses to reviewer comments.docx [file pone.0326312.s003.docx]

**Reviewer’s comments**

**Comments:**

Please review your reference list to ensure that it is complete and correct. If you have cited papers that have been retracted, please include the rationale for doing so in the manuscript text, or remove these references and replace them with relevant current references. Any changes to the reference list should be mentioned in the rebuttal letter that accompanies your revised manuscript. If you need to cite a retracted article, indicate the article has retracted status in the References list and include a citation and full reference for the retraction notice.

**Response:**

We sincerely thank the Editor for the valuable comments and for highlighting important concerns regarding the references. In response, we have conducted a thorough review of all references cited in the manuscript. We confirm that none of the cited articles have been retracted. All references are recent, relevant, and appropriately aligned with the context, rationale, and scientific content of the manuscript. Additionally, we have ensured consistency and accuracy in formatting using Mendeley Reference Manager, adhering to the PLOS ONE citation style. The revised and verified reference list is provided on pages 17–19, lines 532–620.

**Reviewer #2:**

***Comment:***

The higher magnification image in Figure 6b is incorrect. The high magnification image of “NS-siRNA + PGE2” is identical to that of PGE2 + Y27632 in Figure 6a.
Please check the image carefully again.

**Response:**

We thank the reviewer for their valuable observation regarding Figures 6a and 6b. In response, we have carefully revised and corrected the figures. The updated versions accurately reflect the intended data, and no concerns remain regarding their content.
